# Very berry health benefits

**DOI:** 10.1002/fft2.36

**Published:** 2020-08-07

**Authors:** Li-Shu Wang

**Affiliations:** Division of Hematology and Oncology, Department of Medicine, Medical College of Wisconsin, Milwaukee, Wisconsin

Berries are widely appreciated for their abundant phytochemicals and bioactive compounds. As such, consumption of berry fruits has been proven to have a profound beneficial impact on human health and diseases. This special issue collected articles that reported recent advances in berry research. For example, one study reported that the fecal material from mice fed black raspberries increase natural killer cells and CD8^+^ T cells in the fecal recipients. Using three types of cherries, another study reported that different dose ranges of cherry juices induce an overall beneficial modulation of the murine gut microbiota and suggest that amounts of cherries consumed should be carefully chosen in the health and nutritional feeding studies. In addition, anti-tumorigenesis of berry polyphenols, health-promoting effects of Myrtle berries, berries’ effects on *Helicobacter pylori* and gastric cancer, and berry compounds for the development of therapeutics are discussed. It is encouraging that overall findings support the beneficial claims of berries on health from both the preventive and therapeutic points of view.

